# Alu methylation serves as a biomarker for non-invasive diagnosis of glioma

**DOI:** 10.18632/oncotarget.8318

**Published:** 2016-03-23

**Authors:** Jian Chen, Wei Huan, Hao Zuo, Longxiang Zhao, Chuanjun Huang, Xiaojiang Liu, Shiqiang Hou, Jing Qi, Wei Shi

**Affiliations:** ^1^ Department of Neurological Surgery, Affiliated Hospital of Nantong University, Nantong, Jiangsu Province, China; ^2^ Comprehensive Surgical Laboratory, Affiliated Hospital of Nantong University, Nantong, Jiangsu Province, China

**Keywords:** glioma, cell-free DNA, Alu, methylation, liquid chip

## Abstract

Current techniques for diagnosing glioma are invasive and do not accurately predict prognosis. We developed a novel, non-invasive liquid chip assay to diagnose glioma and predict prognosis. Using this method, we determined the methylation state of the Alu element in cell-free DNA extracted from the serum of 109 glioma patients. Controls included 56 patients with benign intracranial tumors and 50 healthy subjects. Matched tumor tissues were processed for 36 patients. The cfDNA from glioma patients showed lower levels of Alu methylation than the controls (P<0.01). Alu methylation was also lower in high-grade than low-grade gliomas (P<0.01), indicating that Alu methylation correlates negatively with disease severity. Moreover, Alu methylation correlated positively with survival (P<0.01). These findings suggest high-throughput liquid chip could serve as a non-invasive diagnostic assay for glioma.

## INTRODUCTION

Gliomas are tumors arising from glial cells and account for almost 70% of primary malignant tumors in the central nervous system. The initial diagnosis for gliomas includes neurologic symptoms and radiographic imaging using CT or MRI [1, 2]. Current imaging techniques do not contribute conclusive diagnostic information and cannot predict prognosis. Thus, the gold-standard to confirm initial diagnosis is a tissue biopsy, which can be problematic due to its highly-invasive nature. Given the highly fatal nature of glioma, early diagnosis is key to a positive prognosis. Consequently, an accurate, efficient and non-invasive technique for early diagnosis is needed to improve the prognosis of glioma patients.

A promising tool for non-invasive diagnostics is cell-free DNA (cfDNA), released by circulating dead or proliferating cancerous cells. Cell-free DNA is increased in patient serum/plasma compared to healthy controls in various types of cancers including lung, colorectal and rheumatoid arthritis cancer [3–10]. Furthermore, cfDNA is increased in peripheral blood samples of cancer patients with tumor metastases. In addition to changes in DNA, the multi-stage and complex nature of cancer also leads to numerous epigenetic alterations. DNA methylation is an important mechanism of epigenetic regulation of gene expression. Specifically, methylation of cfDNA has been linked to cancer initiation and progression [11–13]. Alu is one of the most abundant short interspersed elements (SINEs) in genomic DNA. With more than one million copies, it comprises over 10% of the whole genome. Given to its abundance, Alu is a candidate for a surrogate marker of baseline methylation level for the entire genomic DNA of an organism [14, 15]. Abnormally methylated Alu might decrease genomic stability, thereby instigating tumorigenesis. Indeed, Alu is rich in CpG and universally hypomethylated in tumor cells [16, 17].

**Figure 1:**
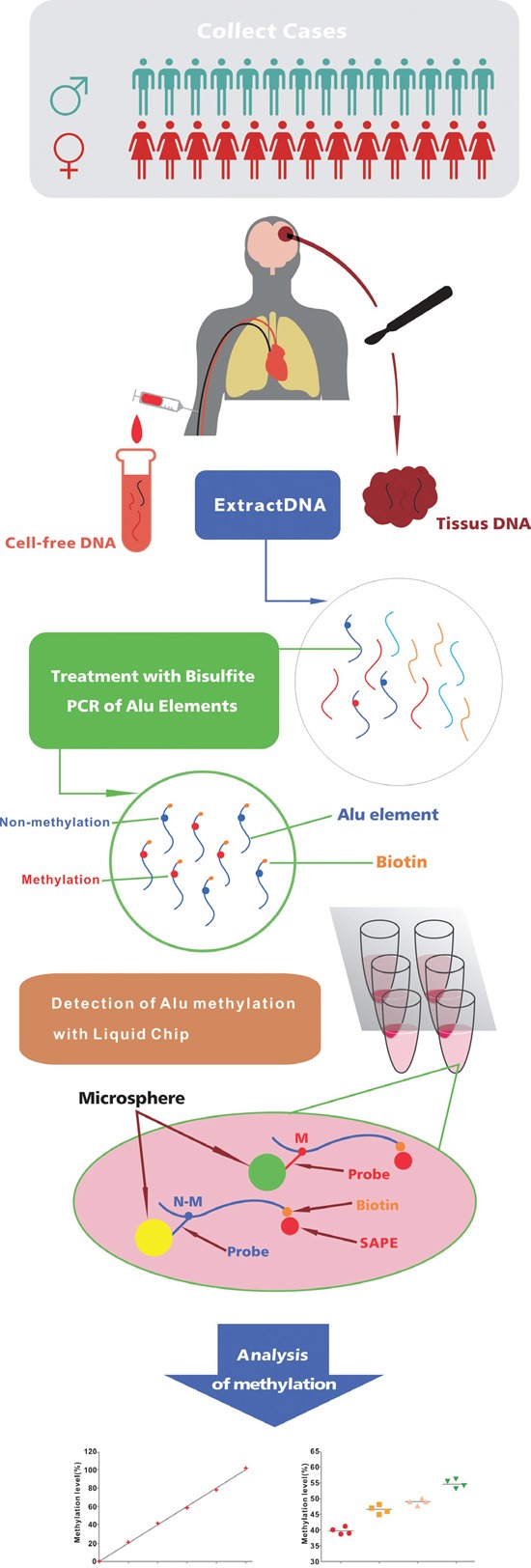
Schematic of study design

We previously reported that the concentration and integrity of Alu in cerebral spinal fluid (CSF) can be used as a biomarker for glioma diagnosis [18]. To extend our previous findings, we tested whether Alu hypomethylation in cfDNA, as measured by a liquid chip system, could serve as a method to detect gliomas. Here we determine the levels of Alu methylation in serum and tumor tissue samples using flow cytometry of microsphere suspension arrays [19](Figure 1). Our data suggest that our non-invasive liquid chip assay can be used in the clinic for non-invasive glioma diagnosis.

## RESULTS

### Alu element is hypomethylated in glioma patients

**Figure 2:**
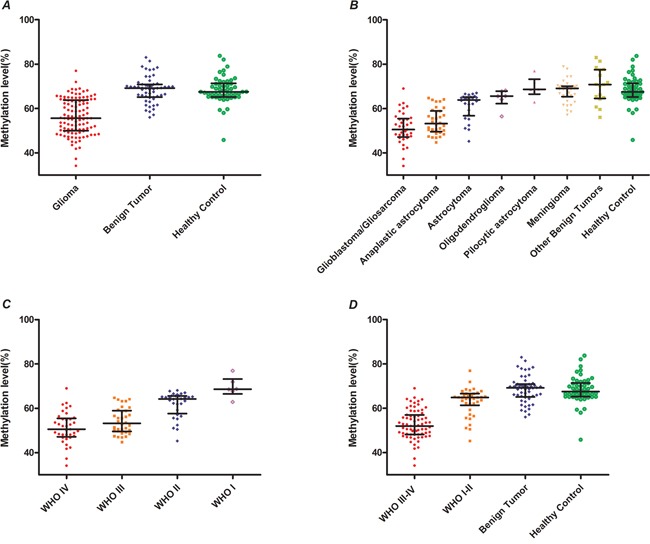
Analysis of Alu methylation levels in cfDNA based on type of glioma **A.** Alu methylation levels in all types of glioma compared to healthy controls. **B.** Comparison of Alu methylation levels in cfDNA between glioma patients, benign and healthy controls. **C.** Comparison of Alu methylation levels in cfDNA among subgroups of glioma patients based on WHO guidelines (WHO I, Pilocytic astrocytoma; WHO II, Astrocytoma and Oligodendroglioma; WHO III, Anaplastic astrocytoma; WHO IV, Glioblastoma and Gliosarcoma). **D.** Comparison of Alu methylation levels in cell-free DNA between high/low grade glioma patients, benign and healthy controls.

We hypothesized that the Alu element was hypomethylated in glioma patients. Methylation levels of Alu repeats were compared across glioma patients, benign controls, and healthy controls. The median methylation levels of Alu were 55.62 % (interquartile range [IQR], 49.98-63.1 %), 69.16 % (IQR, 65.14-70.88 %) and 67.54 % (IQR, 65.25-71.37 %) in glioma patients, benign controls and healthy volunteers, respectively (Figure 2a). Therefore, Alu methylation levels in cfDNA are lower for glioma patients than for both benign and healthy controls (P<0.01).

Next we analyzed Alu methylation levels for each subgroup of glioma patients. Our results indicate lower methylation levels of Alu in glioblastoma and gliosarcoma patients (50.56 %; IQR, 47.16-55.48 %) as well as in anaplastic astrocytoma cases (53.22 %; IQR, 49.62-58.98 %) compared to healthy controls (P<0.01). There was no statistically significant difference in Alu methylation levels between astrocytoma, oligodendroglioma, pilocytic astrocytoma and benign tumor cases and healthy controls (P>0.05; Figure 2b). These results also indicate significantly lower methylation levels of Alu in high-grade gliomas (World Health Organization or WHO grade III-IV) compared to low-grade gliomas (WHO grade I-II) (Figure 2c). Furthermore, the Alu methylation level of high-grade glioma patients (52.01 %; IQR, 48.18-57.02 %) was lower than that for control patients (P<0.01). Finally, there was no statistically significant difference between Alu methylation of cfDNA in low-grade glioma patients and control patients (64.86 %; IQR, 61.28-66.59 %; P>0.05; Figure 2d). These results suggest Alu hypomethylation is associated with the malignancy of gliomas.

### Glioma patient survival correlates positively with Alu methylation levels

**Figure 3:**
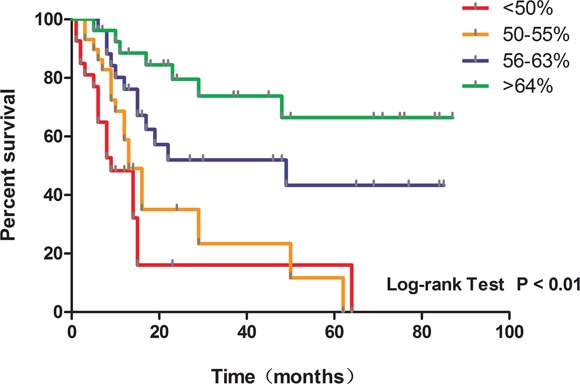
Kaplan-Meier survival curves showing the overall survival in four gradual Alu methylation level groups Cases with Alu methylation level lower than 50% are shown in red, 50-55% in orange, 56-63% in purple and higher than 64% in green.

We assessed the prognostic significance of Alu methylation levels by computing survival curves for the glioma patients in this study. The overall survival of different patient groups with four gradual Alu methylation levels are shown in Kaplan-Meier survival curves (Figure 3). A significant correlation between survival and methylation levels was found by combination analysis (i.e., the longer the overall survival of the patient, the higher the levels of Alu methylation found in patient samples).

### Methylation level of Alu in other different characteristics

**Figure 4:**
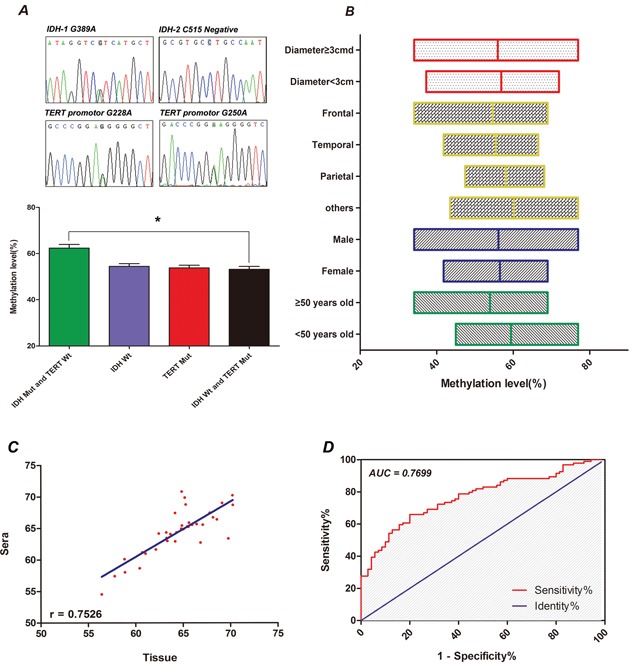
**A.** Chromatograms of sequencing results (top) for IDH-1, IDH-2, and TERT promotor 228, 250. Comparison of Alu methylation levels in cfDNA (bottom) between IDH Mut/TERT WT, IDH1 WT, TERT Mut, and IDH WT/TERT Mut. **B.** Distribution characteristics of Alu methylation level for a number of clinicopathological characteristics. **C.** Correlation of Alu methylation levels between serum and paired tumor tissue samples. **D.** ROC curve of Alu methylation level between glioma patients and controls.

We grouped 61 paired cases into mutant IDH (Mut) and wild-type TERT (WT), IDH WT, TERT Mut, IDH WT and TERT Mut based on IDH-1, IDH-2 and TERT promotor mutations. The median Alu methylation level was 54.19 % (IQR, 48.12-61.55 %) for IDH WT and 54.15 % (IQR, 50.18-58.79 %) for TERT Mut. The IDH WT and TERT Mut group exhibited the lowest median Alu methylation level, 53.52 % (IQR, 49.16-58.70 %); on the other hand, the IDH Mut and TERT WT group had the highest methylation level, 64.21 % (IQR, 58.06-65.46 %). The difference between these two groups was of statistical significance (P=0.0009; Figure 4a). Furthermore, there was no correlation between Alu methylation and gender, or tumor location and size (P>0.05). The median Alu methylation level in patients over 50 years old was slightly lower than in younger patients, but this difference was not statistically significant and may have resulted from characteristics of glioma patient distribution by age (Figure 4b). A correlation analysis was performed between serum samples and tumors of 36 patients (Figure 4c). A significant concordance exists between serum and tumor methylation levels (Spearman r=0.7526; 95 % confidence interval [CI], 0.5564-0.8693; P<0.01).

### Methylation level of Alu in cfDNA is of diagnostic value

To estimate the accuracy of Alu hypomethylation in cfDNA as an early diagnostic indicator for glioma, a receiver operating characteristic (ROC) curve was generated on the basis of methylation levels in glioma patients. The area under the curve was 0.8931 (95 % CI, 0.8509-0.9353; P<0.001; Figure 4d). Consequently, the optimal cutoff value (Youden's index) was 65.03 %, with 84.40 % sensitivity and 80.19 % specificity. These data suggest that Alu hypomethylation in cfDNA serves as a promising biomarker for early glioma diagnosis.

## DISCUSSION

Malignant gliomas are the most common primary cancer of the central nervous system. To date, tumor-derived cfDNA has been evaluated as a potential diagnostic tool for the detection of systemic cancer, making it an important method to diagnose gliomas by blood-based methods. Alu elements, being one of the most abundant SINEs, contribute to genetic evolution, genetic disease and population diversity. Alu elements have been proposed to contribute to disease by two mechanisms: through insertional mutagenesis and non-allelic homologous recombination that induces genetic deletions and duplications [16]. Abundant repeats like Alu elements contribute to the stability of genetic structures. On the other hand, aberrantly methylated Alu elements rich in CpG might influence the expression of downstream genes and result in genetic instability. Tumorigenesis of glioma may rely considerably on aberrant genetic expression and non-integrity. Serum contains a high amount of cfDNA with a low levels of contaminating extraneous DNA released from monocytes[20] as compared to blood, and could therefore serve as a source to study cfDNA biomarkers associated with cancer. A limitation of serum cfDNA as a prognostic tool to study CNS tumors is that nucleic acids cannot easily cross the blood brain barrier. Few studies to date report the analysis of circulating DNA in patients with brain tumors. However, in the present study, we extracted cfDNA from the serum of glioma patient and demonstrated an electrophoretic band indicating Alu element of 247bp after amplification by PCR (Supplement file 2.). This result was consistent across serum and tissue samples. These data suggest that gliomas may shed DNA fragments into the peripheral circulation and that the blood-brain barrier does not prevent cfDNA from being released into bloodstream [21, 22].

Recent techniques developed for detecting DNA methylation (HPLC, BSP, MSP, Methylight etc.) [23] are accurate but complicated or costly. The microsphere arrays utilized in this study work by recognition of 5-hydroxymethylcytosine (hmC) or thymidine in CpG islands (CGIs) of Alu elements after bisulfite conversion. In a matter of hours, the methylation levels of Alu from hundreds of individual specimens were easily measured and available for analysis. Microsphere arrays do not appear to confer any advantage over currently used classical techniques in terms of the qualitative and quantitative assessment of genomic DNA methylation state. However, by simultaneously detecting multiple nucleic acid sequences in each single reaction, microsphere arrays can assess genomic DNA methylation more efficiently and affordably than classical single-reaction methods [24]. Although our primary results need to be confirmed in larger studies, we provide a promising methodology for early clinical detection of gliomas. It is important to note that Alu hypomethylation occurs in diseases other than glioma. Therefore, different diseases including infectious diseases, pregnancy-associated disorders, trauma and systemic cancers may also cause methylation aberrations in DNA, decreasing the reliability of Alu methylation detection for glioma diagnosis. A more-focused approach that examines the methylation status of glioma-specific DNA in peripheral circulation may be necessary.

A significant concordance in Alu methyalation levels between serum and tissue samples demonstrates the utility of this method as a substitute for obtaining DNA from tumor tissues. Importantly, no statistical correlation was identified between the methylation level of Alu and gender, age, tumor location or tumor size in patients. Furthermore, Alu methylation levels were significantly lower in benign tumors than in healthy controls. Alu methylation levels were also lower in high-grade glioma than in low-grade glioma groups. Finally, we found a strong correlation between Alu methylation levels and overall survival. These data suggest that the more severe the glioma, the lower the Alu methylation levels. Numerous genetic studies have shown that glioma patients with wild type IDH or mutations in the TERT promoter have a worse prognosis [25]. Our data was in agreement with these studies. Here we investigated the association of 2 genomic mutations (IDH, TERT promoter) combined with the methylation status of CpG sites #17 and #18 in the Alu element. In future studies, it would be interesting to detect other Alu CpG sites, as well as expand the glioma-specific genic mutations to study. Based on our results, the methylation level of Alu in cfDNA could serve as a high-throughput, non-invasive and reliable prognostic indicator, analogous to a “liquid biopsy”.

## MATERIALS AND METHODS

### Collection of human samples

We obtained peripheral blood samples from 109 glioma patients (71 WHO III-IV, 38 WHO I-II) and 56 benign intracranial tumors patients (39 meningioma, 17 hypophysoma) from the Department of Neurological Surgery, Affiliated Hospital of Nantong University (Nantong, People's Republic of China) (Supplemental file 1.), 36 of which had matched tumor tissues (8 Glioblastoma, 11 Anaplastic astrocytoma 4 Astrocytoma, 3 Oligodendroglioma, 10 meningioma). Patient tumors were characterized as histologically confirmed Glioblastoma (GBM), Gliosarcoma, Anaplastic astrocytoma, Astrocytoma, Oligodendroglioma and other benign tumors (e.g., meningioma, hypophysoma, etc.). Serum was acquired by centrifugation of the peripheral blood at 3,500 rpm for 10 min at room temperature, and stored at −80°C. All tissue samples were collected from patients during resection and frozen and stored immediately −80°C. All blood samples were obtained preoperatively to avoid bias due to iatrogenic injury of the blood-brain barrier.

Control samples were collected from 50 healthy volunteers recruited by the Department of Clinical Laboratory, Affiliated Hospital of Nantong University. The research was conducted according to institutional and ethical guidelines, including the provision of written informed consent for each patient. The study protocol was approved by the Independent Ethics Committee of the Affiliated Hospital of Nantong University.

### DNA extraction from samples and treatment with bisulfite

CfDNA was extracted from serum using a QIAamp MinElute Virus Spin Kit (Qiagen, Dusseldorf, Germany) and tumor tissue DNA was extracted using a QIAamp DNA Mini Kit (Qiagen), according to manufacturer's instructions. Bisulfite treatment of prepared DNA was performed using an Epitect Bisulfite Kit (Qiagen). Non-methylated cytosine in 0.6~1.0 μg (mean 0.8 μg) DNA extracted from serum and tumors was converted to uracil by treating with bisulfate. The product was eluted to an end volume of 20 μl and stored at −20°C.

### PCR of Alu elements

Alu primers were designed according to consensus sequences as previously described [26]. The sequences of the Alu primers are shown in Supplement Table.2. Finally, 50 μl PCR products bonded with biotin were obtained and stored at −20°C (see Supplemental file 2).

### Sequencing for IDH, TERT promotor

Sixty-one paired tumor samples were randomly selected from the glioma patients. We chose two well-known glioma-associated genes, IDH-1 (3417), IDH-2 (3418) and TERT (7015) promoter, for sequencing using *Sanger Sequencing* (Supplemental file 2).

### Detection of methylation level by microsphere arrays

Among the 17 available sites of the Alu element, we designed mated probes to recognize and capture CpG sites #17 and #18 due to ease of design, The biotin-bound Alu elements were subsequently hybridized with streptavidin or R-phycoerythrin (SAPE, Life technologies) coated microspheres. The two types of microspheres were enveloped with probes designed to specifically recognize either methylated or unmethylated Alu elements. The hybridization procedure was carried out in 96-well plates (48 wells in duplicates) away from direct light. Six wells were used to establish the standard curve and one as a negative control, leaving 41 wells available to analyze different samples in each experiment. Alu methylation levels were detected by using a Luminex200 System (Luminex, Austin, Texas, USA). A 635 nm, 10 mW red diode laser was used to excite the fluorochromes contained within the microspheres, while a high-speed digital signal processing distinguished the type of microsphere based on its spectral address and quantified the surface reporter fluorochrome (SAPE), excited by a 532 nm, 13 mW yttrium aluminum garnet (YAG) laser [24]. Thousands of microspheres carrying different Alu elements conjugated with R-phycoerythrin were interrogated, identified, and counted rapidly and accurately (Supplemental file 2.).

### Statistical analysis

All data were analyzed using IBM SPSS statistics (v.21) and GraphPad prism software (v.5). We used two-sample t test to compare methylation levels of Alu in cfDNA from patients vs healthy controls and to determine potential differences in methylation levels between other subgroups of samples. The concordance of methylation status between tumor and serum samples of patients was assessed using the Spearman correlation coefficient. Clinicopathological data were compared by Fisher's exact test, Chi-square, and two-sample t tests. Overall survival (OS), defined as the time from histological diagnosis to death, was determined using the Kaplan -Meier method and the log rank test. A receiver operating characteristic (ROC) curve was generated to assess the validity of using cfDNA methylation levels for glioma diagnosis. In all of these tests, a p value lower than 0.05 was considered significant.

## SUPPLEMENTARY FIGURES AND TABLES




