# Supplementary material for: Alu methylation serves as a biomarker for non-invasive diagnosis of glioma
Source: Oncotarget. 2016 Mar 23;7(18):26099–106. doi: 10.18632/oncotarget.8318 (PMC5041967; doi:10.18632/oncotarget.8318)

SUPPLEMENTARY DATA

Supplementary File S1: Clinical information of patients involved in this study

See Supplementary File S1

Supplementary File S2: Supplement Materials and Methods for in this study

A High Efficiency Approach for the Early Diagnosing Glioma: Rapid Detection of Alu Methylation Level in Cell-free DNA by Liquid Chip

Alu original sequences

GGCCGGGCGCGGTGGCTCACGCCTGTAATC
CCAGCACTTTGGGAGGCCGAGGCGGGCGGATCA
CCTGAGGTCAGGAGTTCGAGACCAGCCTGGCCA
ACATGGTGAAACCCCGTCTCTACTAAAAATACAA
AAATTAGCCGGGCGTGGTGGCGCGCGCCTGTAAT
CCCAGCTACTCGGGAGGCTGAGGCAGGAGAATC
GCTTGAACCCGGGAGGCGGAGGTTGCAGTGAGC
CGAGATCGCGCCACTGCACTCCAGCCTGGGCGAC
AGAGCGAGACTCCGTCTC

24 potential CpG sites were identified by grey shading in Alu original sequences. CpG sites #17 and #18 marked by light yellow shading was the target of the probes designed in our study. The two sequences marked by borders indicate the location of the primers designed.

Part 1. PCR for Alu element

PCR Primers

Alu-F: 5'-biotin-GTTTGTAATTTTAGTATTTTGGGAGGT-3'
Alu-R: 5'-biotin-TCTATCRCCCAAACTAAAATACAATAAC-3'

PCR Reaction System (50 µl)

Bisulfate-converted DNA preparation	5 µl
Forward primer (10 µM)	1 µl
Reverse primer (10 µM)	1 µl
HotStar Taq DNA polymerase	1 µl
dNTP	5 µl
Buffer	4 µl
ddH_2O	33 µl

PCR Reaction Condition

Steps	Temp	Time	
Initial denaturation	94 °C	5 min	
Denature	94 °C	30 s	40 cycles
Anneal	57 °C	30 s	
Extension	72 °C	30 s	
Final extension	72 °C	5 min	

Final PCR products bonded with biotin were stored at −20 °C.

Electrophoresis

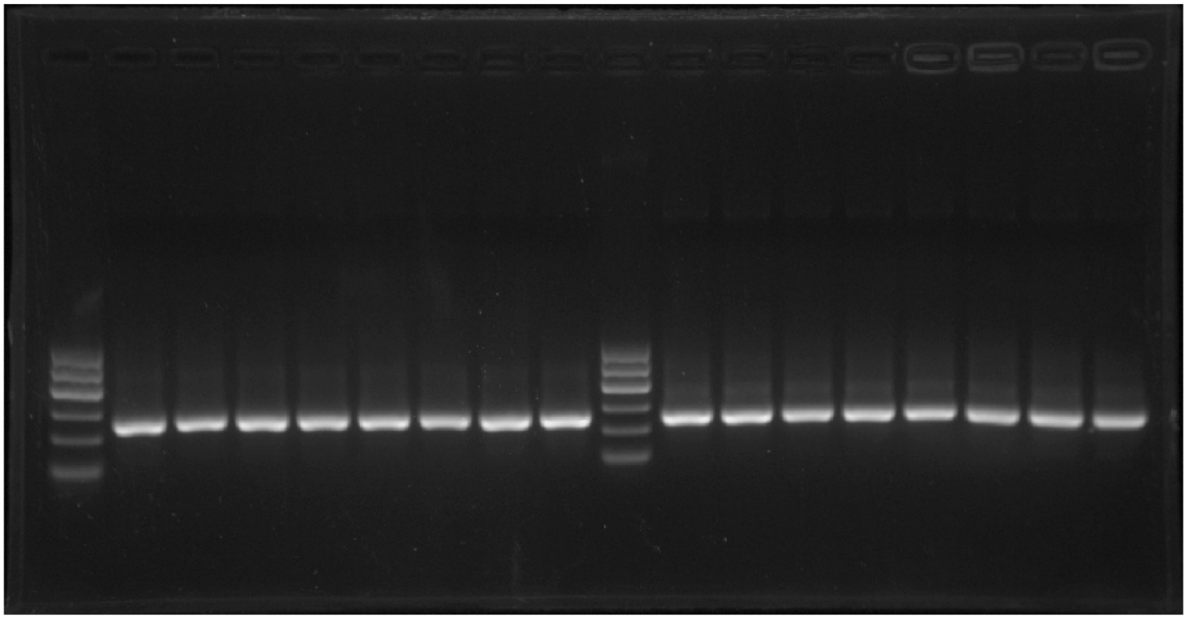

Part 2. Detection of Alu methylation level by microsphere array

Probes aim at CpG #17# and #18 of Alu

Probe-CG: NH2-TTTTTTTTTT GAA TTC GGG AGG CGG AGG T
Probe-TG: NH2-TTTTTTTTTT GAA TTT GGG AGG TGG AGG T
The hybridization procedure was carried out in 96-wells plates (48 in duplicates), while working away from direct light.
Six wells were used to establish the standard curve and one as negative control.
Forty-one different samples can be detected in each experiment.

Hybridization System (100 µl)

Biotin-bound amplified Alu elements		3 µl
Tetramethylammonium chloride solution (TMAC, Sigma)		16.8 µl
1× Tris-EDTA Buffer (TE, Sigma)		5 µl
Microspheres enveloped with probe-CG		0.1 µl
Microspheres enveloped with probe-TG		0.1 µl
SAPE premix	Streptavidin, R-phycoerythrin (SAPE, Life technologies)	0.6 µl
	5µl 1× Tris-EDTA Buffer (TE, Sigma)	74.4 µl

Process of Hybridization

Steps	Temp	Time
Hybridization mixture (25 µl, without SAPE premix)		
Initial denaturation	95 °C	5 min
Hybridization	47 °C	30 min
Add 75µl SAPE premix		
Hybridization	47 °C	15 min

Place the 96-wells plates into Luminex200 System (Luminex, Austin, Texas, USA) as soon as possible, away from direct light.

Part 3. Sequencing for IDH-1 (3417), IDH-2 (3418), TERT (7015)

PCR Primers

IDH-1-F: 5'-ACCAAATGGCACCATACGA-3'
IDH-1-R: 5'-GAATAAAACACATACAAGTTGGAAA-3'
IDH-2-F: 5'-TGCAGAGACAAGAGGATGGC-3'
IDH-2-R: 5'-GCTGAAGAAGATGTGGAAAAGT-3'
TERT-F: 5'-TCTCCGCATGTCGCTGGTT-3'
TERT-R: 5'-ATTCGCGGGCACAGACG-3'

PCR Reaction System (50µl)

Dna-template	1 µl
Forward primer (10 µM)	1 µl
Reverse primer (10 µM)	1 µl
Taq DNA polymerase (5 U/µl)	0.5 µl
dNTP (10 µM)	1 µl
Taq Buffer	5 µl
$MgCl_2$ (25 mM)	5 µl
ddH_2O	35.5 µl

PCR Reaction Condition

Steps	Temp	Time	
Initial denatuatione	95 °C	3 min	
Denature	94 °C	30 s	35 cycles
Anneal	55-60 °C	35 s	
Extension	72 °C	40-50 s	
Final extension	72 °C	8 min	

IDH-1

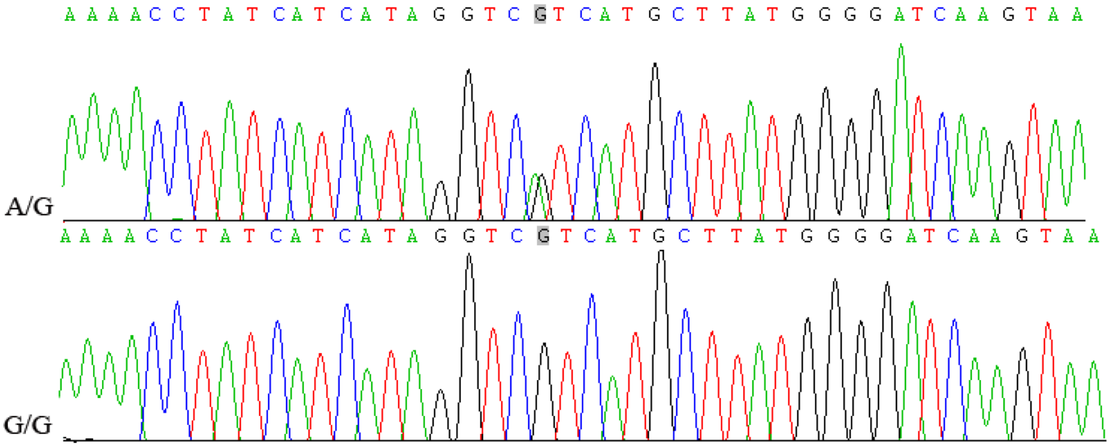
A A A A C C T A T C A T C A T A G G T C G T C A T G C T T A T G G G G A T C A A G T A A
A/G
A A A A C C T A T C A T C A T A G G T C G T C A T G C T T A T G G G G A T C A A G T A A
G/G

IDH-2

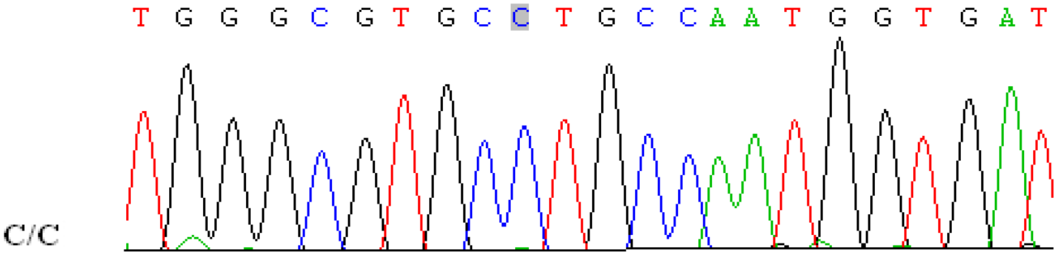
T G G G C G T G C C T G C C A A T G G T G A T
C/C

TERT Promotor (228)

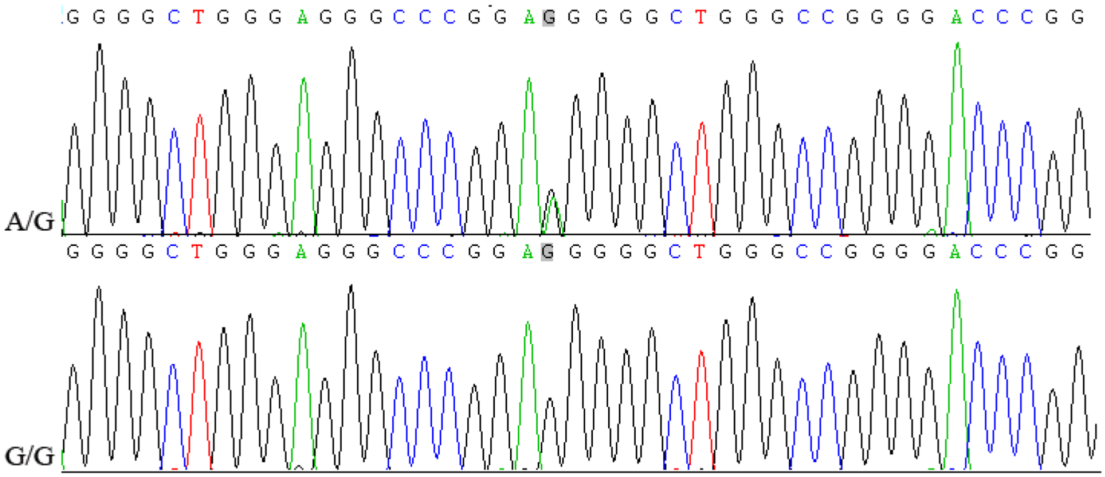

TERT Promotor (250)

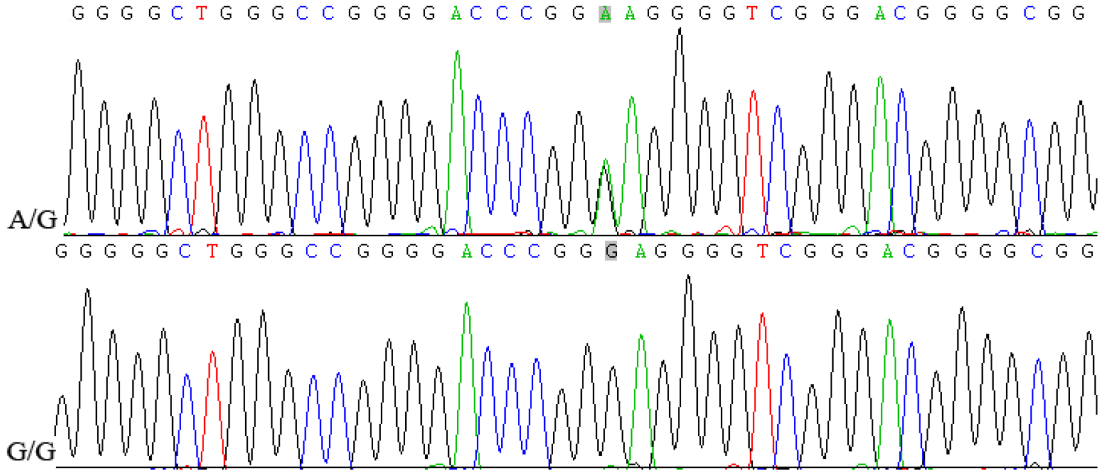

Supplement: Supplementary file 1 [file oncotarget-07-26099-s001.pdf]
