# Supplementary material for: Alu methylation serves as a biomarker for non-invasive diagnosis of glioma
Source: Oncotarget. 2016 Mar 23;7(18):26099–106. doi: 10.18632/oncotarget.8318 (PMC5041967; doi:10.18632/oncotarget.8318)
Supplement: Supplementary file 2 [file oncotarget-07-26099-s002.pdf]

Patient no.	Age/ Gender	Pathology/WHO grade	Location	Size (cm)	Current Status	Methylation Level (%)
**10**	3/M	Pilocytic astrocytoma/WHO I	L Cerebellum	3*3*4	Dead (23 months postoperation)	76.9789
**53**	2/M	Pilocytic astrocytoma/WHO I	R Cerebellum	3*4*1	Dead (10 months postoperation)	71.9592
**72**	51/M	Pilocytic astrocytoma/WHO I	R Frontal,Temporal	2*2*0.5	Survival (18 months postoperation)	68.5692
**100**	20/F	Pilocytic astrocytoma/WHO I	R Cerebellum	3*1*1	Dead (day of operation)	62.8660
**102**	39/M	Pilocytic astrocytoma/WHO I	R Frontal,Temporal	0.5*0.2*0.2	Survival (75 month postoperation)	67.7256
**106**	21/M	Pilocytic astrocytoma/WHO I	Posterior Cranial Fossa	3*2*1	Survival (23 month postoperation)	68.7260
**110**	42/F	Oligodendroglioma/WHO II	L Frontal,Parietal	5*4.5*3	Survival (87 month postoperation)	68.1480
**111**	40/M	Oligodendroglioma/WHO II	L Frontal,Parietal	2*2*1	Survival (50 month postoperation)	65.6103
**112**	37/F	Oligodendroglioma/WHO II	R Frontal	3*3*3	Survival (45 month postoperation)	67.7997
**9**	46/F	Oligodendroglioma/WHO II	R Frontal	7*6*4	Survival (46 months postoperation)	56.4998
**38**	42/M	Oligodendroglioma/WHO II	R Temporal,Occipital	7*5*4	Survival (38 months postoperation)	65.6683
**115**	33/M	Oligodendroglioma/WHO II	R Frontal	9*7*3	Survival (76 month postoperation)	64.1197
**108**	33/F	Astrocytoma/WHO II	R Temporal	7*4*3	Survival (85 month postoperation)	63.6516
**109**	51/M	Astrocytoma/WHO II	L Occipital	4*4*1.8	Survival (84 month postoperation)	65.5400
**122**	48/M	Astrocytoma/WHO II	R Frontal,Parietal	5*9*4	Survival (22 month postoperation)	65.0261
**123**	63/M	Astrocytoma/WHO II	L Temporal	3*4*1.5	Survival (21 month postoperation)	64.8764
**45**	18/F	Astrocytoma/WHO II	R Frontal	7*4*2.5	Survival (37 months postoperation)	64.2984
**67**	63/F	Astrocytoma/WHO II	L Temporal,Parietal	2*1*0.5	Survival (13 months postoperation)	64.1502
**68**	50/M	Astrocytoma/WHO II	R Cerebellum	1*1*0.5	Survival (84 months postoperation)	62.1156
**74**	36/M	Astrocytoma/WHO II	L Frontal	5*3.5*2.5	Survival (83 months postoperation)	64.8530
**77**	74/M	Astrocytoma/WHO II	L Temporal	4.5*3*3	Survival (77 month postoperation)	63.3296
**101**	50/F	Astrocytoma/WHO II	L Temporal	3*3*3	Survival (76 month postoperation)	65.1718
**105**	41/F	Astrocytoma/WHO II	R Frontal,Temporal	8*7*3	Survival (24 month postoperation)	65.2672
**107**	57/M	Astrocytoma/WHO II	L Temporal	4*4*1	Dead (5 months postoperation)	66.5868
**113**	40/F	Astrocytoma/WHO II	L Frontal,Parietal	2.5*3.0*2	Survival (17 month postoperation)	67.0297
**114**	44/F	Astrocytoma/WHO II	L Frontal	4*4*4	Survival (17 month postoperation)	59.2769
**116**	16/F	Astrocytoma/WHO II	Fourth Ventricle	2.5*2*0.8	Dead (29 months postoperation)	66.2273
**117**	29/F	Astrocytoma/WHO II	Saddle Area	4*3*2	Survival (14 month postoperation)	55.6232
**118**	38/M	Astrocytoma/WHO II	R Frontal,Parietal	7*4*3	Survival (71 month postoperation)	66.5867
**119**	68/M	Astrocytoma/WHO II	R Frontal	3*2*1	Dead (13 months postoperation)	55.2373
**120**	20/F	Astrocytoma/WHO II	Fourth Ventricle	2*1.5*1	Survival (65 month postoperation)	61.9433
**124**	38/F	Astrocytoma/WHO II	R Temporal,Parietal	5*4*3	Dead (15 months postoperation)	57.1525
**125**	37/M	Astrocytoma/WHO II	R Temporal	7*6*5	Dead (49 months postoperation)	62.3073

**126**	15/M	Astrocytoma/WHO II	R Lateral Ventricles	2*1*1	Dead (62 months postoperation)	50.4901
**127**	35/M	Astrocytoma/WHO II	R Temporal	8*5*2	Dead (50 months postoperation)	51.0802
**128**	38/F	Astrocytoma/WHO II	L Temporal	8*6*3	Survival (69 month postoperation)	64.9817
**203**	47/M	Astrocytoma/WHO II	L Temporal	4*3*3	Survival (12 month postoperation)	45.2726
**210**	53/F	Astrocytoma/WHO II	R Parietal	5*3*3	Survival (10 month postoperation)	52.4875
**42**	54/F	Anaplastic astrocytoma/WHO III	R Temporal	3*3*1	Dead (17 months postoperation)	64.7957
**46**	57/M	Anaplastic astrocytoma/WHO III	R Frontal	2*1*1	Dead (6 months postoperation)	44.7729
**75**	59/F	Anaplastic astrocytoma/WHO III	R Frontal,Parietal	5*5*4	Survival (16 months postoperation)	60.6871
**83**	49/F	Anaplastic astrocytoma/WHO III	R Frontal	6*6*3	Survival (10 months postoperation)	48.7294
**92**	42/F	Anaplastic astrocytoma/WHO III	R Frontal	7.5*4*2	Survival (16 months postoperation)	50.1680
**131**	47/F	Anaplastic astrocytoma/WHO III	L Frontal	6*5*3	Survival (48 month postoperation)	63.2673
**135**	66/F	Anaplastic astrocytoma/WHO III	R Temporal,Occipital	6*4*2	Dead (15 months postoperation)	47.2402
**138**	67/M	Anaplastic astrocytoma/WHO III	R Temporal	9*7*3	Dead (12 months postoperation)	58.8660
**139**	45/M	Anaplastic astrocytoma/WHO III	R Temporal	5*4*3	Dead (48 months postoperation)	64.1588
**141**	55/M	Anaplastic astrocytoma/WHO III	R Temporal	5.5*5.8*3	Dead (15 months postoperation)	63.2147
**142**	65/F	Anaplastic astrocytoma/WHO III	L Cerebellum	1*1*0.3	Dead (6 months postoperation)	60.4130
**2**	52/M	Anaplastic astrocytoma/WHO III	Saddle Area	6*6*0.5	Dead (19 months postoperation)	58.6987
**15**	65/F	Anaplastic astrocytoma/WHO III	L Parietal	5*5*4	Survival (30 months postoperation)	56.2027
**30**	61/F	Anaplastic astrocytoma/WHO III	R Parietal	3.5*3*1	Survival (27 months postoperation)	63.9649
**32**	58/M	Anaplastic astrocytoma/WHO III	R Temporal	6*5*3	Dead (29 months postoperation)	54.0702
**36**	49/M	Anaplastic astrocytoma/WHO III(recur)	R Frontal,Temporal	3.5*2.5*0.5	Dead (10 months postoperation)	50.5950
**40**	58/M	Anaplastic astrocytoma/WHO III	R Parietal	1*1*0.4	Dead (1 month postoperation)	47.4423
**63**	67/M	Anaplastic astrocytoma/WHO III	R Frontal,Temporal	4*3*1.5	Dead (3 months postoperation)	52.9183
**65**	44/M	Anaplastic astrocytoma/WHO III	L Frontal	5*6*5.5	Dead (12 months postoperation)	55.6207
**69**	35/F	Anaplastic astrocytoma/WHO III(recur)	R Frontal,Parietal	5*2*2	Dead (9 months postoperation)	55.5867
**80**	36/F	Anaplastic astrocytoma/WHO III	L Occipital	5*5*2.5	Survival (13 months postoperation)	57.0222
**81**	40/F	Anaplastic astrocytoma/WHO III	L Frontal	5*5*4	Survival (12 months postoperation)	59.3154
**94**	61/F	Anaplastic astrocytoma/WHO III(recur)	R Temporal	7*5*3	Dead (10 months postoperation)	56.5654
**129**	64/M	Anaplastic astrocytoma/WHO III	L Temporal,Parietal	7*5*2	Dead (9 months postoperation)	52.2574
**130**	71/M	Anaplastic astrocytoma/WHO III	R Temporal,Parietal	6*4.5*2	Dead (13 months postoperation)	50.6137
**133**	76/M	Anaplastic astrocytoma/WHO III	L Temporal	5.5*4.5*2	Dead (5 months postoperation)	47.0431
**136**	33/F	Anaplastic astrocytoma/WHO III	L Temporal	5*5*0.25	Dead (9 months postoperation)	46.9442
**137**	65/M	Anaplastic astrocytoma/WHO III	L Temporal,Occipital	3.5*2.5*1.5	Survival (10 month postoperation)	48.4409
**140**	44/F	Anaplastic astrocytoma/WHO III	L Temporal	4*3*2	Dead (64 month postoperation)	49.2092
**197**	27/M	Anaplastic astrocytoma/WHO III	L Temporal	3*2*1	Survival (14 month postoperation)	51.0688

**198**	66/F	Anaplastic astrocytoma/WHO III	R Temporal	1*1*1	Survival (13 month postoperation)	53.5289
**202**	57/M	Anaplastic astrocytoma/WHO III	L Frontal	4*3*4	Dead (12 months postoperation)	50.4781
**209**	71/M	Anaplastic astrocytoma/WHO III	R Temporal,Parietal	5*2.5*3	Survival (10 month postoperation)	49.7627
**211**	39/F	Anaplastic astrocytoma/WHO III	L Frontal,Temporal	1*1*0.8	Survival (10 month postoperation)	51.4639
**71**	74/M	Glioblastoma/WHO IV	R Frontal	10*5*3	Dead (17 months postoperation)	56.4059
**84**	48/M	Glioblastoma/WHO IV	R Temporal,Occipital	8*7*5	Dead (9 months postoperation)	58.8164
**88**	51/M	Glioblastoma/WHO IV	L Frontal,Temporal	5*5*5	Dead (8 months postoperation)	61.0279
**98**	56/M	Glioblastoma/WHO IV	R Temporal,Occipital	10*6*5	Survival (7 month postoperation)	62.4271
**149**	47/M	Glioblastoma/WHO IV	L Frontal	10*8*5	Dead (8 months postoperation)	57.7814
**150**	66/M	Glioblastoma/WHO IV	L Parietal,Occipital	3.5*2.5*1.5	Survival (23 month postoperation)	47.5211
**151**	56/F	Glioblastoma/WHO IV	R Temporal	5*4*2	Dead (14 months postoperation)	46.7722
**156**	64/M	Glioblastoma/WHO IV	L Temporal	3*5*8	Survival (10 month postoperation)	50.5600
**37**	68/M	Glioblastoma/WHO IV(recur)	R Frontal	5*3*2	Dead (9 months postoperation)	55.7947
**41**	60/F	Glioblastoma/WHO IV	L Occipital	8*6*4	Dead (8 months postoperation)	43.5172
**43**	54/F	Glioblastoma/WHO IV(recur)	L Parietal,Occipital	4*3*2	Survival (24 months postoperation)	52.7280
**47**	59/M	Glioblastoma/WHO IV	R Frontal,Temporal	4*3*2	Dead (1 month postoperation)	37.2762
**51**	61/F	Glioblastoma/WHO IV	L Frontal	5*6*6	Dead (11 months postoperation)	69.0183
**54**	57/M	Glioblastoma/WHO IV	L Frontal,Temporal	6*4.5*1.5	Dead (2 months postoperation)	48.0886
**55**	57/M	Glioblastoma/WHO IV	R Temporal	12*12*4	Dead (22 months postoperation)	61.7175
**59**	50/M	Glioblastoma/WHO IV	R Temporal	4*4*2	Dead (16 months postoperation)	53.5235
**61**	69/F	Glioblastoma/WHO IV	L Temporal	8*3*2	Dead (8 months postoperation)	41.7908
**62**	61/F	Glioblastoma/WHO IV	R Temporal,Parietal	5*3*1.5	Dead (15 months postoperation)	48.1769
**73**	62/F	Glioblastoma/WHO IV(recur)	L Occipital	7.5*5*4	Dead (16 months postoperation)	52.8707
**76**	50/M	Glioblastoma/WHO IV(recur)	R Temporal	6*6*3	Dead (6 months postoperation)	44.9923
**86**	77/M	Glioblastoma/WHO IV	R Temporal	7*6*3	Dead (5 months postoperation)	53.6104
**87**	65/M	Glioblastoma/WHO IV	L Frontal,Parietal	6*6*5	Dead (3 months postoperation)	55.0949
**89**	42/M	Gliosarcoma/WHO IV(recur)	R Temporal	3.5*3.5*3.5	Dead (7 months postoperation)	55.1610
**144**	58/M	Glioblastoma/WHO IV	R Parietal	3*3*1.5	Survival (69 month postoperation)	60.2081
**146**	65/M	Glioblastoma/WHO IV	R Frontal	4*3.5*2	Dead (2 months postoperation)	49.1582
**147**	51/F	Glioblastoma/WHO IV	L Frontal	4*3*2.5	Dead (14 months postoperation)	48.1005
**152**	54/F	Glioblastoma/WHO IV	R Temporal	3.8*2*1	Dead (6 months postoperation)	50.1968
**153**	41/F	Glioblastoma/WHO IV	L Frontal	5*5*2	Survival (10 month postoperation)	52.0093
**154**	75/M	Glioblastoma/WHO IV	L Frontal	8*2*7	Dead (3 months postoperation)	34.1077
**212**	49/M	Glioblastoma/WHO IV	R Temporal	4*4*3	Survival (9 month postoperation)	54.7669
**213**	55/M	Glioblastoma/WHO IV	L Frontal	3*2*2	Survival (9 month postoperation)	51.9683

**216**	73/F	Glioblastoma/WHO IV	L Frontal,Temporal	3*5*2	Dead (8 months postoperation)	46.7918
**219**	67/M	Glioblastoma/WHO IV	R Temporal	6*5*5	Dead (6 months postoperation)	42.2831
**220**	69/F	Glioblastoma/WHO IV	L Lateral Ventricles	6*8*8	Survival (9 month postoperation)	48.4191
**223**	54/M	Glioblastoma/WHO IV	R Frontal	4*3*3	Survival (8 month postoperation)	46.1477
**224**	72/M	Glioblastoma/WHO IV	R Temporal	2*2*3	Survival (3 month postoperation)	49.7822
**226**	59/F	Glioblastoma/WHO IV	L Frontal	2*5*3	Survival (1 month postoperation)	48.8702
**157**	62/M	Meningioma	——	——	——	65.9390
**164**	57/F	Meningioma	——	——	——	66.4282
**165**	35/M	Meningioma	——	——	——	66.8222
**166**	56/F	Meningioma	——	——	——	70.2067
**172**	50/F	Meningioma	——	——	——	67.0703
**174**	67/M	Meningioma	——	——	——	70.2398
**175**	59/F	Meningioma	——	——	——	68.5510
**177**	58/M	Meningioma	——	——	——	69.1077
**178**	50/M	Meningioma	——	——	——	65.4283
**187**	72/F	Meningioma	——	——	——	69.7663
**158**	53/M	Meningioma	——	——	——	65.0408
**159**	53/F	Meningioma	——	——	——	70.3970
**160**	45/F	Meningioma	——	——	——	69.1374
**161**	44/F	Meningioma	——	——	——	69.2928
**162**	76/F	Meningioma	——	——	——	62.9930
**163**	64/F	Meningioma	——	——	——	63.3781
**167**	63/F	Meningioma	——	——	——	69.6718
**168**	45/F	Meningioma	——	——	——	68.7041
**169**	56/M	Meningioma	——	——	——	69.1831
**170**	57/M	Meningioma	——	——	——	65.9237
**171**	62/F	Meningioma	——	——	——	70.0869
**173**	75/M	Meningioma	——	——	——	67.4732
**176**	60/F	Meningioma	——	——	——	68.6209
**179**	46/F	Meningioma	——	——	——	61.4629
**199**	55/M	Meningioma	——	——	——	58.5762
**200**	65/M	Meningioma	——	——	——	57.1368
**3**	49/F	Meningioma	——	——	——	61.5954
**5**	74/M	Meningioma	——	——	——	74.4506
**13**	63/F	Meningioma	——	——	——	74.3856

**19**	76/F	Meningioma	——	——	——	72.5334
**25**	44/M	Meningioma	——	——	——	69.9047
**28**	61/F	Meningioma	——	——	——	77.9361
**31**	53/F	Meningioma	——	——	——	78.9318
**35**	60/M	Meningioma	——	——	——	69.7809
**52**	47/M	Meningioma	——	——	——	71.2218
**56**	45/M	Meningioma	——	——	——	59.0629
**60**	71/F	Meningioma	——	——	——	61.2565
**79**	56/F	Meningioma	——	——	——	69.7034
**85**	48/M	Meningioma	——	——	——	69.7288
**180**	64/M	Hypophysoma	——	——	——	78.5097
**181**	58/M	Hypophysoma	——	——	——	77.4186
**182**	80/F	Hypophysoma	——	——	——	70.8879
**183**	55/M	Hypophysoma	——	——	——	71.7641
**184**	44/M	Hypophysoma	——	——	——	75.0057
**185**	60/M	Hypophysoma	——	——	——	59.6088
**186**	43/F	Hypophysoma	——	——	——	81.3920
**155**	61/M	Hypophysoma	——	——	——	65.4648
**188**	51/M	Schwannoma	——	——	——	70.8649
**189**	22/F	Schwannoma	——	——	——	66.1298
**190**	17/M	Schwannoma	——	——	——	64.9092
**191**	49/F	Schwannoma	——	——	——	83.0309
**192**	62/M	Schwannoma	——	——	——	56.0348
**193**	53/F	Cyst	——	——	——	77.6566
**194**	30/M	Cyst	——	——	——	70.4655
**195**	8/M	Cyst	——	——	——	64.3267
**196**	57/M	Cyst	——	——	——	60.7043
